# Revealing the Molecular Mechanisms of Alzheimer’s Disease Based on Network Analysis

**DOI:** 10.3390/ijms222111556

**Published:** 2021-10-26

**Authors:** Abdulahad Bayraktar, Simon Lam, Ozlem Altay, Xiangyu Li, Meng Yuan, Cheng Zhang, Muhammad Arif, Hasan Turkez, Mathias Uhlén, Saeed Shoaie, Adil Mardinoglu

**Affiliations:** 1 Centre for Host-Microbiome Interactions, Faculty of Dentistry, Oral & Craniofacial Sciences, King’s College London, London SE1 9RT, UK; abdulahad.bayraktar@kcl.ac.uk (A.B.); simon.1.lam@kcl.ac.uk (S.L.); saeed.shoaie@scilifelab.se (S.S.); 2 Science for Life Laboratory, KTH–Royal Institute of Technology, SE-17121 Stockholm, Sweden; havva.altay@scilifelab.se (O.A.); xiangyu.li@scilifelab.se (X.L.); meng.yuan@scilifelab.se (M.Y.); cheng.zhang@scilifelab.se (C.Z.); muhammad.arif@scilifelab.se (M.A.); mathias.uhlen@scilifelab.se (M.U.); 3 Department of Medical Biology, Faculty of Medicine, Ataturk University, Erzurum 25240, Turkey; hasanturkez@yahoo.com

**Keywords:** Alzheimer’s disease, gene co-expression network, genome-scale metabolic model, reporter metabolite analysis, energy metabolism

## Abstract

The complex pathology of Alzheimer’s disease (AD) emphasises the need for comprehensive modelling of the disease, which may lead to the development of efficient treatment strategies. To address this challenge, we analysed transcriptome data of post-mortem human brain samples of healthy elders and individuals with late-onset AD from the Religious Orders Study and Rush Memory and Aging Project (ROSMAP) and Mayo Clinic (MayoRNAseq) studies in the AMP-AD consortium. In this context, we conducted several bioinformatics and systems medicine analyses including the construction of AD-specific co-expression networks and genome-scale metabolic modelling of the brain in AD patients to identify key genes, metabolites and pathways involved in the progression of AD. We identified *AMIGO1* and *GRPRASP2* as examples of commonly altered marker genes in AD patients. Moreover, we found alterations in energy metabolism, represented by reduced oxidative phosphorylation and ATPase activity, as well as the depletion of hexanoyl-CoA, pentanoyl-CoA, (2E)-hexenoyl-CoA and numerous other unsaturated fatty acids in the brain. We also observed that neuroprotective metabolites (e.g., vitamins, retinoids and unsaturated fatty acids) tend to be depleted in the AD brain, while neurotoxic metabolites (e.g., β-alanine, bilirubin) were more abundant. In summary, we systematically revealed the key genes and pathways related to the progression of AD, gained insight into the crucial mechanisms of AD and identified some possible targets that could be used in the treatment of AD.

## 1. Introduction

**Figure 1:**
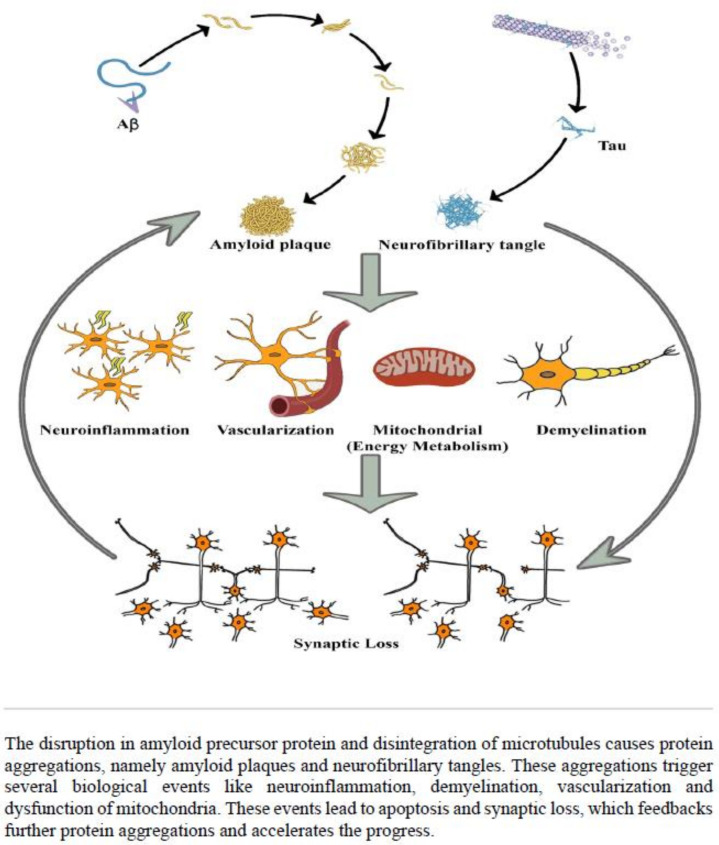
Systematic representation of the pathophysiology of Alzheimer’s disease.

Alzheimer’s disease (AD) is the most frequently diagnosed neurodegenerative disorder worldwide and the sixth leading cause of death in the United States and is on the rise [1]. The disorder is characterised by amyloid plaques and neurofibrillary tangles; cell loss, vascular damage and dementia follow as a direct result of the vicious cycle of their deposition (Figure 1) [2]. Along with age and family history, inheritance also plays an essential role in the development of AD [3]. Among several genetic risk factors, the *APP*, *PSEN1*, *PSEN2* had been identified as a causative factor for early-onset AD, the APOE-ε4 allele (encodes a protein that transports cholesterol in the bloodstream) was shown to have a strong impact on late-onset AD. However, studies based on APOE status among different racial and ethnic groups have shown inconsistent results [1,4,5]. Despite a number of treatments being approved by the U.S. Food and Drug Administration (FDA), none of those therapeutic strategies can cure the disease [6]. There is strong evidence that early diagnosis and treatment might help to decelerate the progression of the disease and maintain brain function [7]. Therefore, rational development of medical approaches (e.g., sophisticated brain imaging studies and discovery of novel candidate genes, proteins, and other substances in blood or cerebrospinal fluid) are fundamental for better understanding of the molecular factors that contribute to disease progression, and for improving the early diagnosis and treatment decisions.

Systems biology-based methodologies have proven effective in providing biological insight into genetic elements and in identifying the most promising biomarkers associated with complex diseases [8,9,10]. For instance, weighted gene co-expression network analysis (WGCNA) was successfully used to construct gene networks in various diseases and identify centrally connected hub genes as promising biomarkers or therapeutic targets [11,12]. Additionally, the use of WGCNA can provide a deeper understanding of which functional regulators may be driving transcriptional signatures in the development of disease, such as transcription factors (TFs) [13]. Moreover, GEnome-scale metabolic Models (GEMs) provide quantitative information on how the different metabolites are linked to each gene and each reaction in the network [8,14,15]. GEMs condense all information about the known associations of protein-encoding genes and how these genes/proteins interact with other bioactive compounds and associated reactions.

**Figure 2:**
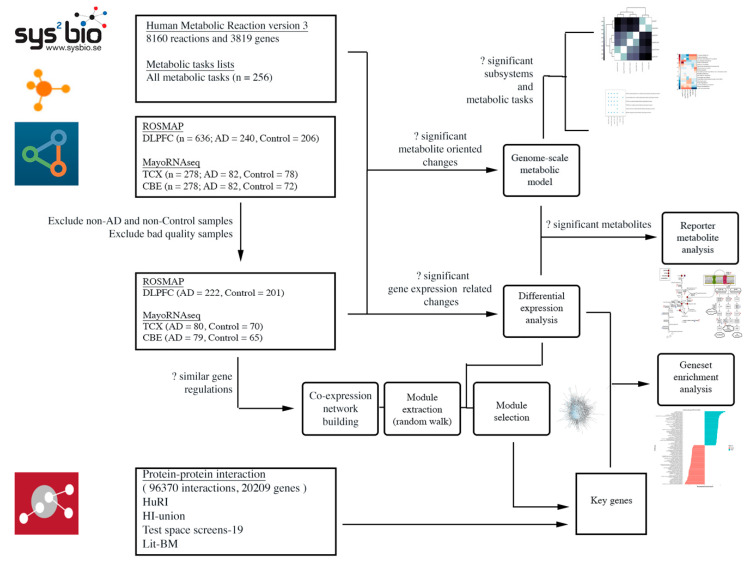
Workflow diagram.

In the present study, we first conducted differential expression analysis (DEA) and gene set enrichment analyses (GSEA) to collect baseline biological information. Second, we constructed gene co-expression networks for each brain region and searched for biologically relevant modules to identify key genes. We evaluated the importance of these key genes with respect to literature, baseline biological information and protein–protein interactions (PPIs). Finally, we reconstructed AD-specific GEMs and performed analysis to depict reporter metabolites and reveal the changes in metabolic pathways. A workflow for this study is presented in Figure 2.

## 2. Results

### 2.1. Distinct Gene Expression and Functional Profiles in Different Brain Regions of AD

We performed DEA to characterise regional gene expression changes in AD compared to control. We found 2885 differentially expressed genes (DEGs) (1472 up, 1413 down) in dorsolateral later prefrontal cortex (DLPFC), 477 DEGs (205 up, 272 down) in temporal cortex (TCX) and 1515 DEGs (944 up, 571 down) in cerebellum (CBE) regions (Supplementary Dataset S2). The DEGs between each pair of regions were significantly overlapped as showed by the significance of shared DEGs (Table 1), and most of these genes were changed in the same direction (Supplementary Dataset S2). Furthermore, 34 genes were found to be significantly differentially expressed in all three datasets. Interestingly, 33 of these 34 genes were found to be expressed in the same direction and involved in many pathways ranging from secretion to signalling and to cancer-associated pathways (Table 2). We then performed Kyoto Encyclopedia of Genes and Genomes (KEGG) [16] pathways enrichment analysis for the 34 genes as well as overlapped genes between each pair of DEGs from these three datasets to investigate their functional relevance. As a result, we found that these overlapped DEGs are over-represented in many cancer-associated pathways, signalling pathways, cell differentiation and apoptosis (Table 3), suggesting a potential link to cell senescence and cell death.

**Figure 3:**
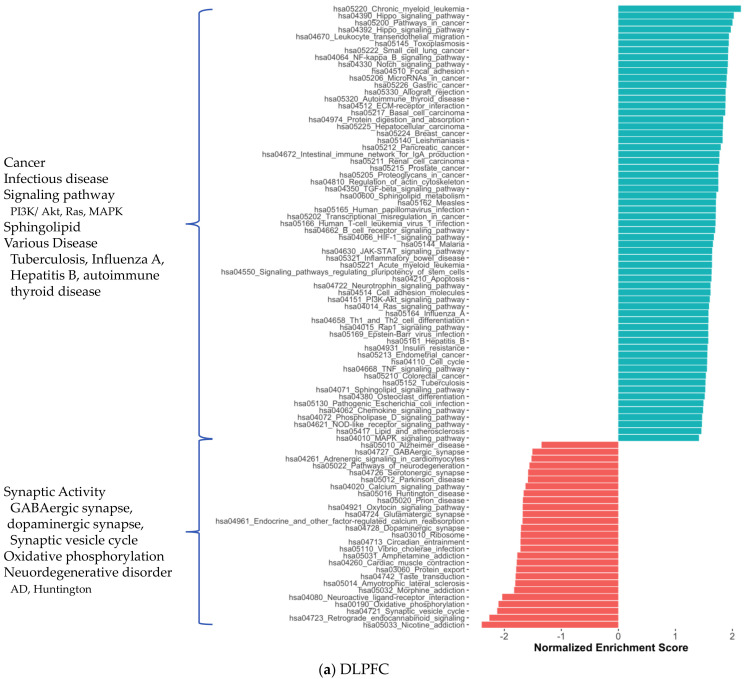

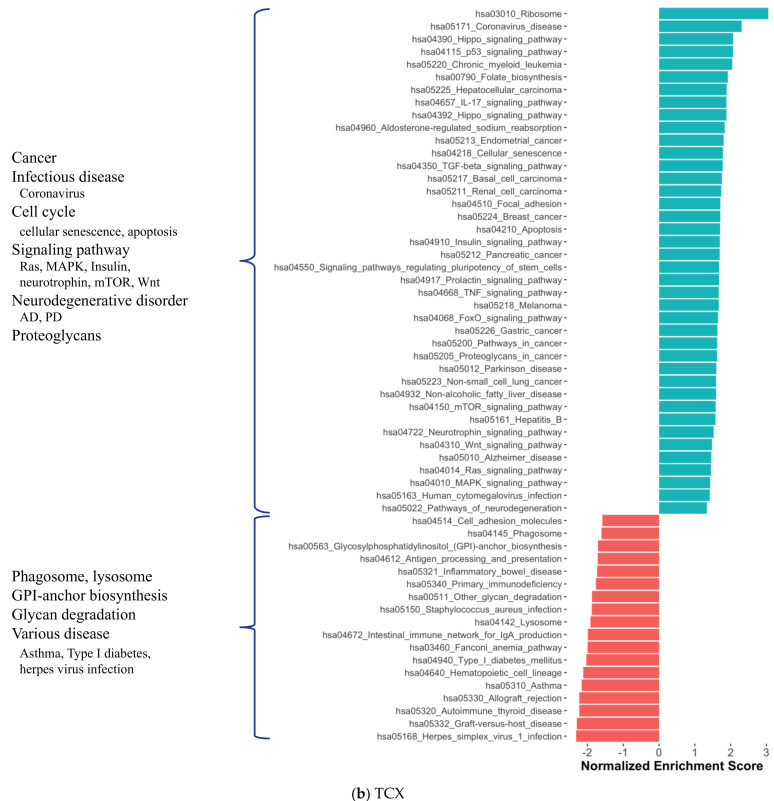

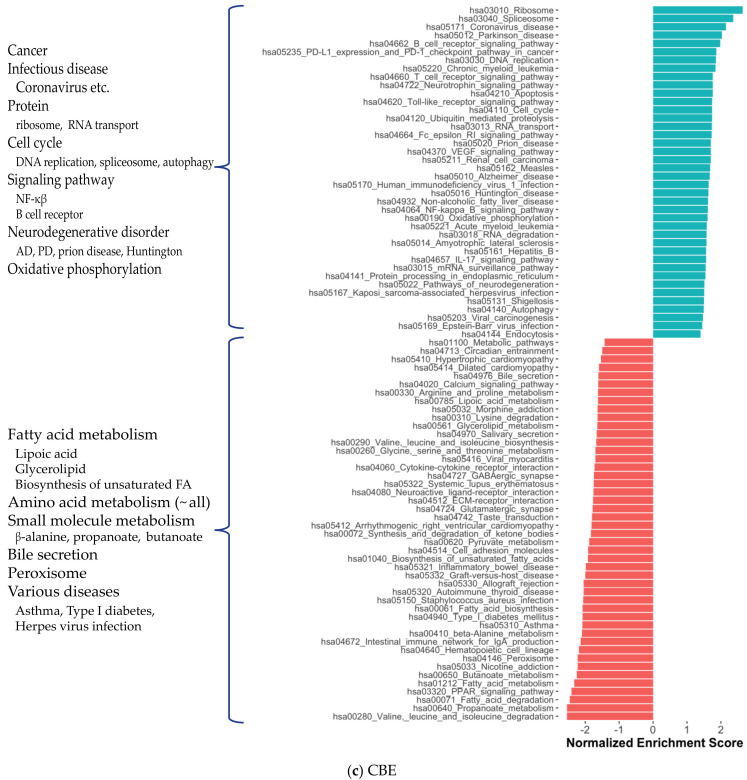
Significantly enriched KEGG pathways for protein-coding genes in DLPFC, TCX and CBE.

We also investigated the functional profiles of DEGs from each brain region using the KEGG pathway and Gene Ontology (GO) [17] term enrichment analysis (Figure 3). Most generally, we found that the cancer pathways were enriched in DEGs of all three regions, while anabolic reactions, e.g., oxidative phosphorylation and amino acid biosynthesis, and synaptic activity tended to diminish. As we mentioned above, all shared DEGs were associated with many pathways enriched with DEGs from brain tissues (Figure S5a,b). The reduction in GABAergic and glutamatergic synapse pathways in CBE is consistent with our knowledge of the GABAergic and glutamatergic neuron loss in AD. We observed reduced oxidative phosphorylation, small molecule metabolism (pyruvate, butanoate, propanoate, beta-alanine, fatty acid) and amino acid degradation (valine, leucine, isoleucine, lysine) that reflects crucial AD-associated changes in brain metabolism. Supported by the literature, the enrichment of HIV-1 infection [18,19] and hepatitis B infection [20] associated pathways, NF-κβ [21,22], VEGF [23] and toll-like receptor signalling [24] pathways, diminishment of peroxisome [25,26] and morphine addiction [27] associated pathways in CBE was found among interesting AD-associated abnormalities. We also observed a reduction in the retrograde endocannabinoid signalling pathway [28] and nicotine addiction [29] associated pathway in DLPFC and CBE. All significant KEGG enrichments are presented in Supplementary Dataset S3.

In parallel, DLPFC and CBE showed abundant GO term enrichment. Cytoskeleton organisation (e.g., actin cytoskeleton organisation), GTPase activity, cell membrane–linked signalling pathways and associated processes (e.g., G protein-coupled receptor signalling pathway, neurotransmitter transport, glutamate receptor signalling pathway) and angiogenesis (e.g., blood vessel morphogenesis, epithelial cell differentiation) were enriched significantly in DLPFC data. We also found the enrichment for cytoskeleton, neuronal regions and respective molecular activity (e.g., microtubule-binding, cadherin binding, Rho GTPase binding) (Figure S6a–c). These enriched pathways indicate changes supporting cholinergic signalling and vascularisation. The enriched pathways in the CBE dataset were similar but scarce compared to DLPFC results (Figure S6d–f). GO enrichment analysis did not yield any significant results for TCX data. 

### 2.2. Co-Expression Network Analysis in Different Brain Regions of AD

**Figure 4:**
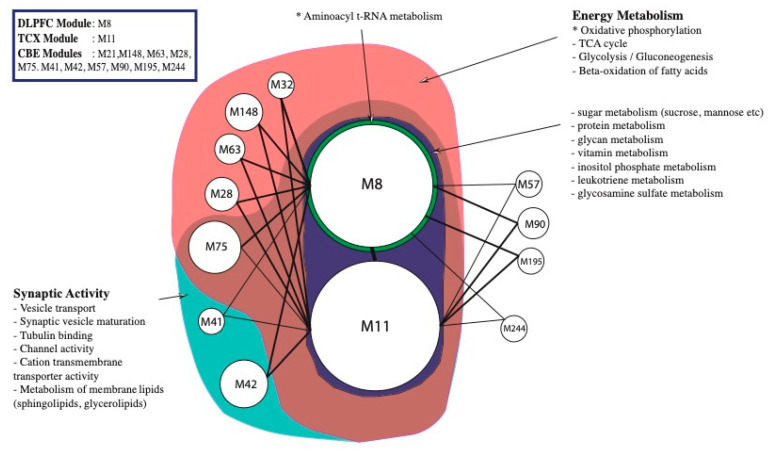
Functional enrichment of significant modules determined from co-expression network by random walk algorithm. M8 (n = 2034), the single module from DLPFC, and M11 (n = 2422), the single module from TCX, were enriched for nearly all metabolic pathways partly due to their size. For instance, genes involved in vitamin, glycan and leukotriene metabolisms were abundant specifically for M8 and M11. Nevertheless, oxidative phosphorylation was the only significant enrichment for both modules (hypergeometric p-value 0.00034 and 0.00054). Aminoacyl t-RNA metabolism was enriched significantly (hypergeometric p-value < 0.0476) for M8 genes. Whereas some CBE modules (M57, M90, M195 and M244) were not enriched for any given annotation, others shared genes associated with synaptic activity and energy metabolism.

We also constructed region-specific co-expression networks to investigate functional gene modules (See methods). We identified one large module for DLPFC (node size = 2034), one large module for TCX (node size = 2422) and 11 modules for CBE (total node size = 574) complying given size and connectivity criteria. Even though the centrality measurement was not as dramatic as in DLPFC and TCX modules (Supplementary Dataset S4), the 11 modules for CBE were merged as one module gene group for analyses hereafter as they have high connectivity (Supplementary Dataset S5). We also observed significant and consistent overlap between modules from different datasets (Supplementary Dataset S5). Then we investigated the association between the DEGs that we identified in different brain regions and the genes involved in each module. We found that the identified modules were overlapped with the down-regulated DEGs from their respective region, suggesting that down-regulated genes in these regions follow a similar regulation pattern. We also generated a merged set of down-regulated genes (n = 1975) which were significant in at least one dataset based on DEA for studying AD changes in general. We found that each module contains nearly one-third of all identified down-regulated DEGs (609 in DLPFC module, 798 in TCX module, 184 in merged CBE module) and a modest amount of these genes were hub genes in the modules (86 in DLPFC module, 89 in TCX module, 17 in merged CBE module). Modules that we determined were then annotated for a list of numerous biological processes and molecular functions, ranging from amino acid biosynthesis to vesicular transport. We classified these annotations into energy metabolism and synaptic activity as shown in the figures (Figure 4, Figure S7a–i). 

We then investigated the status of genes related to amyloid and tau hypotheses in modules and differential expression analysis. These are comprised of genes responsible for amyloid precursor protein (APP) synthesis and catabolism down to Aβ (i.e., *APP*, *BACE1*, *PSEN1*, *PSEN2*, *NCSTN*, *ADH1* and *ADAM10*) and tau synthesis (i.e., *MAPT*). Only two genes, *APP* and *PSEN2*, were found in our modules, DLPFC and TCX, and they were down-regulated in these datasets. Despite not being in the modules, significantly changed expression of *MAPT* (down) and *ADAM10* (up) in DLPFC were noteworthy. 

We detected two hub genes shared in DLPFC, TCX and CBE modules: *GPRASP2* and *AMIGO1*. *GPRASP2* is a G-protein coupled receptor (GPCR) regulator. It facilitates endocytosis of GPCRs from the plasma membrane to lysosomes for degradation [30]. *AMIGO1* is important for the growth of neurites and may contribute to the myelination of neural axons [31]. *AMIGO1* and *GPRASP1,* which come from the same protein family of *GPRASP2*, are significantly down-regulated in DLPFC. Notably, *GPRASP2* is also down-regulated with bolder line significance (adjusted *p*-value in DLPFC = 0.0568). According to Brain Atlas data on Human Protein Atlas (HPA) [32], *GPRASP2* has relatively high expression levels in brain tissues overall compared to other tissues (https://www.proteinatlas.org/ENSG00000158301-GPRASP2/tissue. Accessed 5 May 2021). There is a 10–15% decrease in the expression of *GPRASP2* in all datasets. We also observed 5% down-regulation in DLPFC, a 10% decrease in CBE and nearly no change in TCX for *AMIGO1*. Still, these small changes may be critical considering that *AMIGO1*’s highest expression is in the cerebral cortex and cerebellum among all tissues (https://www.proteinatlas.org/ENSG00000181754-AMIGO1/tissue. Accessed 5 May 2021).

Moreover, other shared hub genes among two datasets include *PAK1* in CBE and DLPFC, *ATP6V1E1*, *CDC42*, *DCTN2*, *ERLEC1* and *MPP1* in CBE and TCX, and 86 other genes between DLPFC and TCX. These genes are mostly associated with glucose-dependent energy metabolism, ubiquitin-proteasome system (UPS), synaptic activity and plasticity, cytoskeleton organisation as well as intra-golgi and retrograde golgi to endoplasmic reticulum traffic.

### 2.3. Alterations in Energy Metabolism, Chaperones and Synaptic Activity 

Based on the network analysis and DEA results, we found a wide change in energy metabolism, chaperones and synaptic activity. Regarding the energy metabolism, we found a batch of ATPase genes that are responsible for ATP catalysis and solute carrier family genes which are responsible for the transport of ATP and its substrates through membranes were affected. *ATP1B3* encodes non-catalytic β-3 subunit of ATPase Na^+^/K^+^ transporting enzyme, which is one of the shared DEGs and expressed 15–40% higher in all AD tissues. *ATPAF1*, which encodes ATP synthase mitochondrial F1 complex assembly factor, and 15 ATPase cation transporting subunit encoding genes (mostly H^+^, one Na^+/^K^+^ and one plasma membrane Ca^+2^ transporting), were significantly down-regulated in DLPFC. *ATP1B2*, *ATP1A3*, *ATP13A4* and *ATP6V0C* were significantly up-regulated in CBE. There were no significant expression changes in ATPases in TCX other than *ATP1B3.* The expression of the hub genes *ATP6V1A* and *ATP6V1B2* shared by DLPFC and TCX*,* and *ATP6V1E1* shared by DLPFC and CBE were not significantly changed. Solute carrier family member genes *SLC9A6, SLC25A4* and *SLC25A14* were shared by the DLPFC and TCX, but their expression was not significantly changed (Table 4). In addition, we found some solute carrier genes showed different expression patterns associated with AD. *SLC25A30*, *SLC7A2* and *SLCO4A1* were up-regulated in DLPFC and TCX; *SLC6A12*, *SLC38A2* and *SLC9A3R2* were up-regulated and *SLC16A6* was down-regulated in DLPFC and CBE; *SLC9A3* was down-regulated in TCX and CBE (Supplementary Dataset S2). These results suggest that the expression of many genes associated with energy production were changed in AD and these genes were mostly up-regulated.

Chaperone proteins, also known as heat shock proteins (HSPs), are crucial for proteostasis. Chaperones can be divided into two large families in eukaryotes: the Hsp70 family (gene symbols: HSPs) and the Hsp40 family (gene symbols: DNAJs). According to our findings, *HSPA12A* and *DNAJC5* showed higher centrality in DLPFC and TCX than other HSPs (Table 4). Seven members of the DNAJ C subfamily were significantly down-regulated in DLPFC but not in TCX. The other three DNAJ genes were significantly up-regulated in CBE. Seven members of HSPs had changes in DLPFC, whereas three other HSP genes were significantly up-regulated in TCX. There are few interactions between HSPs and other proteins. *DNAJC30* encodes a mitochondrial protein that is enriched in neurons and regulates mitochondrial respiration. This protein interacts with a mitochondrial rRNA methyltransferase encoded by *MRM1*, which has lower expression in DLPFC AD samples. It was reported that *MRM1* catalysed methyltransferase which is important for the mitoribosome assembly and synthesis of electron transport chain submits [33]. In addition, our findings show us an interaction between *DNAJC30* and *RNF170* proteins. *RNF170* was down-regulated in all tissues, especially in TCX. A strong association of *RNF170* and active inositol 1,4,5-trisphosphate (IP3) receptors—the former mediates the ubiquitination of the latter—was shown in an animal proteomics study [34]. IP3 receptors are located on the endoplasmic reticulum as calcium channels and are degraded by UPS upon activation. *HSP90B1* interacts with *SGTB* on the protein level, which is a co-chaperone and regulates Hsp70 ATPase activity. Both *HSP90B1* and *SGTB* were down-regulated in DLPFC. 

We also observed a wide dysregulation of synapse-associated genes in AD. The results showed that more than a third of synaptic genes were differentially altered (mostly down-regulated) in DLPFC and CBE tissues for each synapse type. Of 27 genes shared in all synapse types, two genes, *GNG12* and *GNGT2*, were up-regulated in DLPFC, while seven genes, namely *GNG2*, *GNG3*, *GNG4*, *GNB5*, *CACNA1D*, *PRKACA* and *PRKCG*, were down-regulated. *GNB4* and *CACNA1D* were down-regulated in CBE. We found eight synaptic activity-associated genes (*AP2M1*, *ARHGEF9*, *CALM3*, *DLG3, GABRB3*, *L1CAM, NPTN* and *NSF*) which interact with other DEGs on protein level and these genes were shared in DLPFC and TCX modules. Most of these genes were down-regulated in DLPFC except for *L1CAM* and *NSF*. *AP2M1* encodes a subunit of assembly protein complex 2, which is crucial for the acidification of endosomes and lysosomes through proton pumping. *CALM3* encodes calmodulin, which is critical for synaptic activity since it mediates voltage-dependent calcium channels [35,36]. Calmodulin also takes part in a protein kinase complex, CaMKIIα, and is critical for learning and memory [37]. The protein encodes by *DLG3* interacts with NMDA receptor subunits at the synapse; thus, it is required for synaptic plasticity and learning [38]. General down-regulation of synaptic activity associated genes both in DLPFC and CBE implies that the cholinergic hypothesis is more plausible to explain AD in our case. *CREB3*, a member of cholinergic and dopaminergic synapses is significantly up-regulated in TCX.

### 2.4. Metabolic Alterations in Different Brain Regions of AD

**Figure 5:**
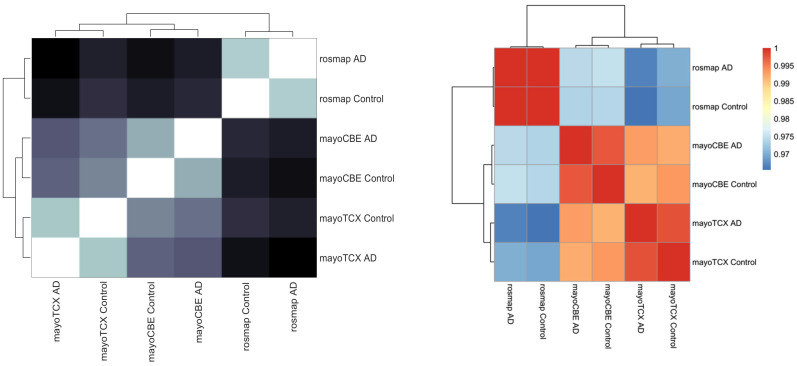
Group comparisons in terms of reaction content and gene expressions. (left) Comparison of GEMs based on reaction content showed in heatmap of Hamming distances and dendrogram. (right) Comparison of groups based on gene expressions showed in heatmap of Spearman correlations of mean TPMs and dendrogram.

**Figure 6:**
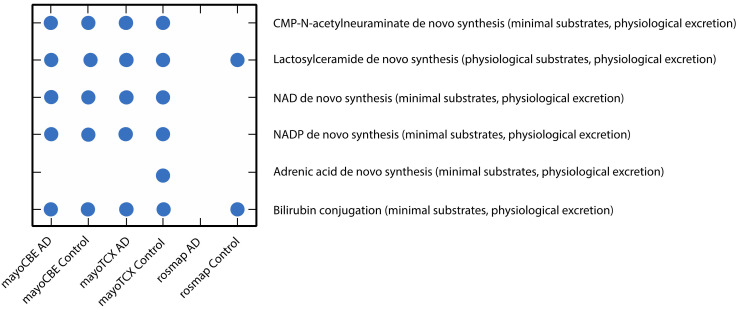
Scatter plot of metabolic tasks succeeded or failed differently in at least one GEM.

**Figure 7:**
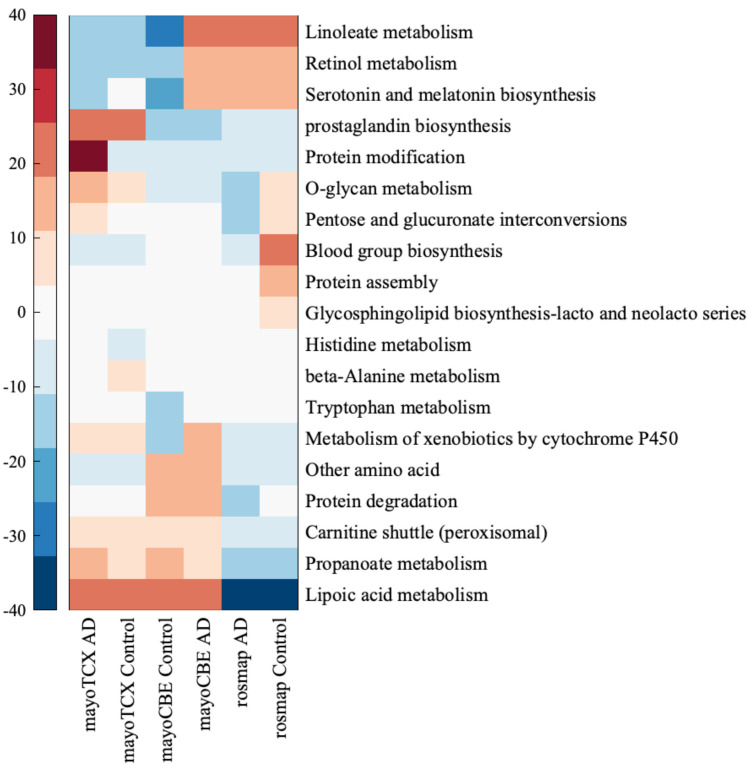
Heatmap for metabolic pathways expressed at least 10% differently in one group of samples. Each colour tone closer to blood-red refers to 10% increase compared to other metabolic pathways, while each colour tone closer to deep blue 10% decrease compared to other metabolic pathways.

To investigate the metabolic alterations in different regions in AD, we performed the GEM analyses. First, we reconstructed the region-specific GEM based on the transcriptomic profiles of each region in the brain. Numbers of reactions, metabolites and genes for each GEM are given in Table 5. Region-specific GEM construction allows us to understand the changes in the activity of metabolites, reactions, subsystems/pathways and metabolic functions/tasks. In terms of reactions, all groups showed a great similarity (≥95%); however, groups from the same brain region compared to other groups had a higher similarity. Group correlations based on gene expressions supported this finding (Figure 5). DLPFC groups were more distinct from others, as we observed in previous analyses. We analysed the metabolic tasks (Figure 6) and the differentially active pathways (Figure 7) by comparing GEMs to enhance our understanding of functional changes in AD from plausible metabolite perturbations. Metabolic tasks were mostly different in DLPFC. Lactosylceramide de novo biosynthesis (a class of glycosphingolipid) and bilirubin conjugation failed in the AD DLPFC group, while they were modelled to be performed successfully in the control DLPFC group. NAD, NADP, adrenic acid and CMP-N-acetylneuraminate (CMP-Neu5Ac) de novo biosynthesis failed in both DLPFC groups. Adrenic acid biosynthesis also failed in other groups apart from the control TCX group; hence, its failure is more likely linked to aging rather than AD [39].

Regional differences in subsystem activities were remarkable. A drastic decrease in lacto-glycosphingolipid biosynthesis, lipoic acid metabolism, O-glycan metabolism, pentose and glucuronate interconversions, blood group biosynthesis and protein assembly were exclusively founded in the DLPFC group. Interestingly, O-glycan metabolism and pentose and glucuronate interconversions were increased in TCX but decreased in DLPFC. In addition, we observed a weak increase in propanoate metabolism, histidine metabolism and protein modification and a decrease in β-alanine metabolism and serotonin and melatonin biosynthesis in TCX. Different from other tissues, CBE samples showed higher activity of linoleate, retinol, xenobiotics and tryptophan metabolisms. In addition, serotonin and melatonin biosynthesis and propanoate metabolism were decreased in CBE but increased in TCX.

**Figure 8:**
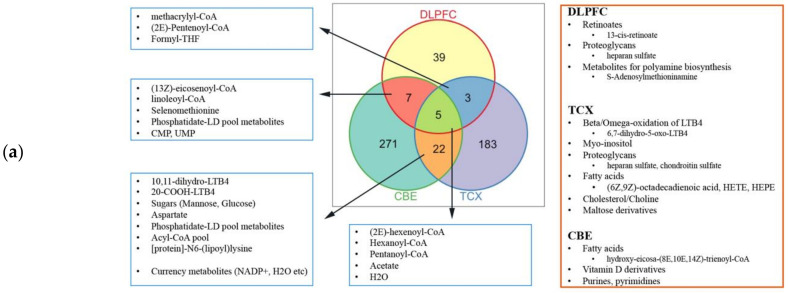

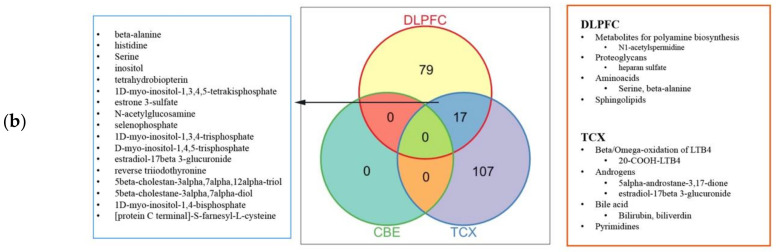
Reporter metabolites for: (**a**) down-regulated genes; and (**b**) up-regulated genes.

Then we performed the report metabolites analysis based on the result of differentially expressed analysis (Datasets S6–8). With the use of only down-regulated genes, we observed the significant changes in the three medium-chain fatty acids (pentanoyl-CoA, hexanoyl-CoA, (2E)-hexenoyl-CoA), acetate and H_2_O for all tissues (Figure 8). We observed that fatty acids were abundant in control groups for all three tissues, suggesting a general decrease in fatty acids including adrenic acid. The lower abundance of (R)-3-hydroxybutanoate in DLPFC was noteworthy, since its importance for feeding neurons from astrocytes. Histidine was reported based on the up-regulated genes in TCX, which aligns with its lower metabolism. Retinoates were reported based on the down-regulated genes in DLPFC. Cholesterol, choline and maltose derivatives were reported based on the down-regulated genes in TCX. Vitamin D derivatives and metabolites responsible for the oxidation of leukotriene B4 (LTB4) were reported based on the down-regulated genes in CBE. LTB associated metabolites were reported based on the up- and down-regulated genes in TCX, implying an interplay. Similarly, proteoglycans were reported based on the up and down-regulated genes in DLPFC. Proteoglycan enrichments were opposing (enriched hsa05205_Proteoglycans_in_cancer and decreased O-glycan metabolism), suggesting that they may cover a different set of genes. CMP and CMP-Neu5Ac were reported based on the down-regulated genes in DLPFC, while CMP-Neu5Ac synthesis showed to be failed in the tissue.

## 3. Discussion

We performed network analysis to reveal the key genes and metabolites involved in the progression of AD. We found that shared DEGs (e.g., ATP1B3, RAF1 and STAT5B), hub genes (GPRASP2 and AMIGO1) and reporter metabolites (pentanoyl-CoA, hexanoyl-CoA, (2E)-hexenoyl-CoA and acetate) associated with AD progression are involved in signalling and energy metabolism. For instance, lower levels of unsaturated fatty acids were observed together with reduced activity of ATPases. Fatty acids enter into beta-oxidation, converted smaller acyl-CoA molecules, such as pentanoyl-CoA, in hepatocyte (liver) and astrocyte (brain). This continues down to the acetyl-CoA and 3-hydroxybutyrate which can diffuse into neurons. In neurons, they are not only used in energy production but also induce BDNF indirectly that promotes synaptic plasticity and stress resistance [40]. We also found that genes encoding ATPases, synaptic activity and plasticity, cytoskeleton organisation, vesicular transport (including endocytosis) and HSPs were tended to be down-regulated. Taken together with the depletion of unsaturated fatty acids and repression in energy metabolism may be affected by or cause synaptic loss and demyelination.

**Figure 9:**
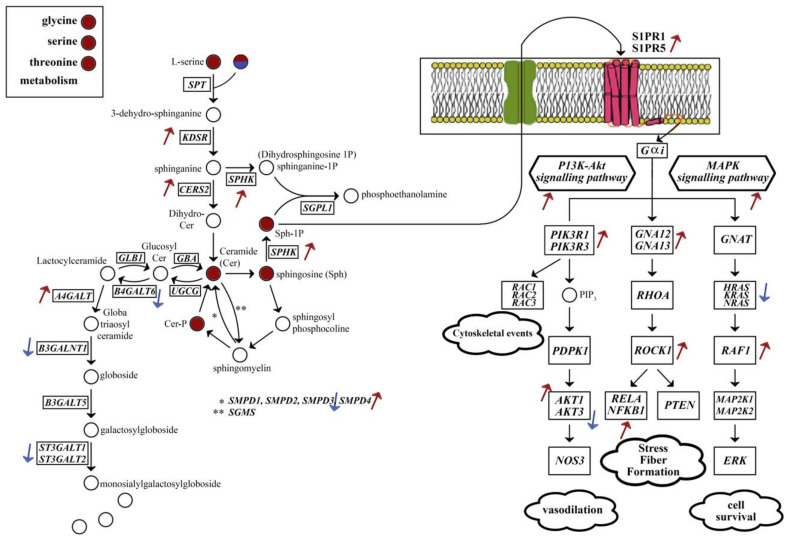
Sphingolipid biosynthesis and affected signalling pathways in DLPFC. There is an overall increase in sphingolipid synthesis. The increase in sphingosine-1-phosphate also induces MAPK and P13K/Akt signalling pathways, which are associated with cytoskeletal events, vasodilation, fibre formation and cell survival. Circles representing metabolites: red for up-regulated genes, red/blue is palmitoyl–CoA reported for both down-regulated and up-regulated genes. Boxes representing reaction catalysing genes. Arrows represent the direction of significant gene expression level change.

Myelination is becoming more central in our understanding of AD and aging, although studies have converged different opinions. One opinion is that AD only comes after the completion of myelination. Another claims that myelin damage precedes amyloid plaque deposition, becomes the first neuropathological abnormality in AD; even more, A*β* and tau proteins may be produced during myelin repair [41]. Brain sphingolipids are being considered important in AD due to the large conformational changes of Aβ in the presence of glycosphingolipids, synthesised from lactosylceramides, possibly ending in Aβ aggregation. In addition to them, membrane microdomains rich in cholesterol, ceramides and glycosphingolipids may control APP processing [42,43]. Sphingolipid metabolism and sphingolipid signalling pathway were found to be enriched in AD DLPFC. Ceramide, ceramide-1-phosphate, sphingosine, sphingosine-1-phosphate and D-gal-N-acylsphingosine were reported for this region for up-regulated genes (Figure 9). We can conclude an overall increase in these metabolites due to up-regulation of *CERS2*, *KDSR* and *SPHK*. The increase in sphingosine-1-phosphate and its receptors on the cellular membrane (*S1PR1* and *S1PR5*) may induce the up-regulation of MAPK and PI3K/Akt signalling pathways. On the other hand, the down-regulation of *B4GALT6* and *B3GALNT1* may create a bottleneck for glycosphingolipid biosynthesis from lactosylceramides and nucleotide sugars, e.g., CMP-Neu5Ac (Figure 3a and Figure 7 for corresponding enrichments). In the case of myelination, the down-regulation of the hub gene *AMIGO1* is also critical. *AMIGO1* expression is specific to the central nervous system and reduced in a pro-inflammatory environment, whereas its isomers are expressed more widely. It is promoted by neuronal growth-promoting factor amphoterin and has shown to be effective for dendritic growth. AMIGO1 was shown to be down-regulated in the post-mortem multiple sclerosis brain as well [44].

The unsolved question is thus how amyloid-β (Aβ) is related to brain degeneration. The modern perspective indicates the interplay between protein aggregation and other events that we provided evidence of. Aβ oligomers can trigger mitochondrial dysfunction and synaptic impairment, in three ways. First, they bind to the cellular form of prion protein on lipid rafts on post-synaptic neuron; this activates N-methyl-d-aspartate receptors (NMDARs), causing Ca^2+^ overload. Ca^2+^ overload triggers neurofibrillary tangle formation, mitochondrial dysfunction, leading to synaptic impairment and then apoptosis [45]. Second, oligomers interact with several NMDAR-inducing membrane receptors, such as EphB2 (Ephrin type-B receptor 2) and α7nAChr (acetylcholine receptor), leading to proteasomal degradation and NMDAR-induced synaptic impairment. Ca^2+^ overload may occur in a third way by binding a specific form of Aβ to Na^+^/K^+^-ATPase α3 and its activation of voltage-gated Ca^2+^ channels (VGCC) [46]. Supporting the expected low levels of *Aβ due to the* diminishment of APP processing genes, NMDAR genes (i.e., GRINA and GRIN2A in DLPFC), VGCC genes (i.e., CACNA2D1, CACNA1D, CACNA1E, CACNA1G), many ATPases, also DLG3 and CALM3, which mediate NMDARs and VGCCs respectively, were down-regulated. Possibly, HSPs were down-regulated in response to a decrease in protein aggregation. Considering these, the mechanisms of Ca^2+^ overload in neurons should be lower.

Interestingly, we found no significant expressional changes for Aβ processing genes *despite* the tendency of lower expression of MAPT, APP and downstream APP processing genes in three brain regions other than underexpression of PSEN2 and overexpression of ADAM10 in DLPFC. While changes in DLPFC were more striking, there were counter-intuitive points beginning with APP processing. The shared hub gene GPRASP2 plays an important role in APP processing as well. GPRASP2 encodes GPCR-associated sorting proteins (GPRASPs). These proteins interact with a broad spectrum of GPCRs and facilitate their degradation. Taken together, explaining the association between lower GPRASP2 expression and amyloid processing depends on further studies about the functionality and interactions of GPRASP2.

Another noteworthy aspect of our findings was related to cancer pathways. The antagonist relationship between cancer and degeneration diseases in aging including cardiovascular and neurodegenerative disorders is intriguing. The accumulating evidence from transcriptomics and genomics put forth an aging-driven trade-off between them; the loss of proteostasis, epigenetic changes and immune-response associated responses mostly occur in a proliferative way in tumour cells, whereas they occur in a way that shortens the lifespan of aged cells. Genomic instability, the secretion of proinflammatory cytokines and deregulated nutrient sensing, which interferes with insulin, mTOR and AMPK signalling pathways, are common to both paths [47,48,49]. In the case of AD, the trade-off was observed at the population level. Retrospective and long-term prospective cohort studies have demonstrated clearly the lower probability of AD among cancer survivors and the lower probability of cancer in AD patients [49,50]. The up-regulation of many cancer-associated genes contributes to the overall enrichment, which plays a decisive role in cell fate. As given in Table 3, *RAF1*, *SMAD4*, *RALBLP1* and *STAT5B* were up-regulated in both cortical regions and cerebellum. Firstly, *RAF1* is a master regulator for cell fate decisions, such as proliferation, differentiation and apoptosis. It can activate the MAPK signalling pathway and may promote cell survival [51]. *SMAD4* induced inhibition of TGF-β activity suppresses tumour activity [52]. In addition, TGF-β signalling pathway activation is important for synaptic transmission; therefore, high *SMAD4* activity may be linked to the synaptic deficit as well [53]. *RALBP1* is challenging to interpret; cause high *RALPB1* activity is considered important for metastasis [54], while its depletion may lead to mitochondrial dysfunction and synaptic deficits [55]. Finally, *STAT5B* responds to cytokines and affects the transcription of transcription factors, such as FOXP3, CD25 and Bcl-2. These factors may induce cell development and prevent apoptosis, depending on the activated tissues [56]. In addition to cancer-associated genes, we find evidence for the switch towards degeneration in metabolic findings. Cancer cells are well known for their high energy and monomer consumption rates [57]. The diminishment in oxidative phosphorylation, which was reflected by lower ATPase expressions, and amino acid and fatty acid biosynthesis lowers the possibility of cancer co-occurrence, whereas the enrichment in signalling pathways explains the high enrichment in cancer pathways.

We assessed some metabolites from reporter metabolite analysis because of their neuroinflammatory or neuroprotective features. Adrenic acid is one of the most abundant fatty acids in the early human brain and is critical for inflammatory response together with arachidonic acid. Arachidonic acid is catalysed to hydroxy-peroxy-eicosatetraenoic acids (HPETEs) and hydroxy-eicosatetraenoic acids (HETEs), which are metabolised into leukotrienes. Leukotrienes have possible inflammatory effects; LTB4 may also enhance Aβ production by modulating γ-secretase activity [58]. On the other hand, it was shown adrenic acid inhibits LTB4 production by neutrophils and also enhances macrophage phagocytosis [59]. Here, we observed that both AD up-regulated and down-regulated genes contribute to the production of leukotrienes, HETEs and HPETEs in TCX and CBE. This may emphasise brain damage and neuroinflammation in the aging brain in general [60,61]. On the other hand, the poor levels of adrenic acid may show the ineffective response of the brain against AD-caused inflammation. Retinoids resemble adrenic acid in this regard. They are vitamin A derivatives that are present abundantly in the hippocampus, cortex and other brain regions. They inhibit LTB production by oxidation, thus, have pro-inflammatory effects. Additionally, they enhance choline acetyltransferase activity; therefore, they are beneficial for acetylcholine production and neurogenesis [60]. Reporter metabolite analysis (RMA) showed a decrease in retinoates in DLPFC, while retinol metabolism increased in CBE. Therefore, it is safer to assume that brain regions have different preferences for the use or production of these metabolites. Other vitamins and vitamin derivatives were mentioned for their neuroprotective effects in previous studies and interplay with fatty acids [62,63,64,65,66].

Propanoate and its products are another group of metabolites that have neuroprotective value. Excessive production of propionyl-CoA, due to high levels of propanoate in the gut, was shown to have neurotoxic effects such as mitochondrial dysfunction, neuroinflammation, glutamate excitotoxicity and decrease in serotonin levels. Propanoate metabolism is also linked to β-alanine metabolism and TCA cycle. Propionyl-CoA is metabolised to malonate semialdehyde, which is converted to acetyl-CoA and is interconverted to β-alanine by transaminase activity and involvement of ketone bodies and amino acids [67]. β-alanine is known to be neurotoxic [68]. It is also reported by both DLPFC and TCX up-regulated genes for this pathway. Propanoate metabolism was found to be more active in the AD TCX group compared to its control, which was represented by the conversion of 3-oxopropionyl-CoA to 3-hydroxypropionyl-CoA in TCX. The lower activity of serotonin biosynthesis in AD is also consistent. In contrast, the propanoate pathway was repressed and similarly, serotonin biosynthesis increased in the AD group of CBE. In addition, more metabolites for this pathway were reported for CBE, e.g., malonyl-CoA. Nevertheless, no DEGs were recorded for this pathway.

Another group of metabolites is bile acids. We observed AD selective failure of bilirubin conjugation in DLPFC. Bilirubin and biliverdin were also found in TCX up-regulated genes. Bilirubin conjugation is the detoxification of degraded bilirubin with glucuronic acid, which is followed by its secretion. Bile acids facilitate dietary fat digestion and were linked to AD through their importance in cholesterol homeostasis and lipid absorption [68,69].

It seemed that many metabolites were severely affected by AD. Among them, unsaturated fatty acids (especially hexanoyl-CoA and pentanoyl-CoA), retinoids and other vitamins are known to improve cognitive abilities [68,69,70,71]. Their neuroprotective effects were studied extensively and put forth clinically [64,65,66]. Thus, reintroducing them may ameliorate cognitive decline. For instance, oral administration of a mixture including L-serine and L-carnitine tartrate has recently been shown to be effective at improving cognitive functions in AD patients [72]. In this regard, further studies are required to test the effects of metabolites that we found against cognitive decline.

In conclusion, we performed co-expression network analysis and metabolic modelling, and applied reporter metabolite analysis. Robustness of methods, analysis of a large number of samples and the use of different brain regions enhanced the reliability of our findings. In summary, we found a decline in energy production, myelination and synaptic activity in all brain regions. We also proposed some of the key genes as biomarkers. Among them, we found that *AMIGO1* and *GPRASP2* are highly critical for AD progression, and these genes should be investigated for their association to other pathological markers in large cohorts.

## 4. Materials and Methods

### 4.1. Data Sources and Sample Selection

Transcriptomic data (i.e., transcript abundances, transcript per million (TPM) values and metadata) of the Religious Orders Study and the Memory and Aging Project (ROSMAP) and Mayo Clinic RNAseq Study (MayoRNAseq) were obtained from the Accelerating Medicines Partnership-Alzheimer’s Disease (AMP-AD) database (https://www.nia.nih.gov/research/amp-ad. Accessed 24 March 2021). Specimens in the ROSMAP study originated from DLPFC, while specimens in the MayoRNAseq study were from TCX and CBE, mostly from the same postmortem individuals. In the ROSMAP dataset, we excluded samples of MCI and other conditions contributing to cognitive impairment in order to remove potential confounders arising from other dementias; therefore, in total 423 samples (AD = 222, control = 201) were included (Supplementary Dataset S1). In MayoRNAseq dataset, TCX and CBE samples were obtained as matching samples from the same individuals. Similarly, we excluded samples of individuals with pathological aging or progressive supranuclear palsy. In total 150 samples (AD = 80, control = 70) for TCX and 144 samples (AD = 79, control = 65) for CBE were selected. For the sake of clarity in writing, ROSMAP dataset is abbreviated as DLPFC, and MayoRNAseq datasets are abbreviated as either TCX or CBE throughout the article. 

### 4.2. Differential Expression Analysis and Gene Set Enrichment Analysis

The *biomaRt* package was used to access gene symbol and accession IDs from ENSEMBL. Only transcripts of protein-coding genes were evaluated. Genes with low expressions (sum of TPM of a gene lower than 1) both in AD and control groups were discarded. The limma [73] *removeBatchEffect* function was used to remove batch effect by using flow cell number for DLPFC, source for TCX and both for CBE as covariates by inspecting top 1000 genes by variance on UMAP plots [74]. The effect of removing these covariates was visible on the plots (Figures S1a-f, S2a-I and S3-f). Differential expression analyses (DEAs) were performed using DESeq2 [75] “parametric” model, which was showed to be fit for mean of normalized gene counts (Figure S4), and adding respective batch covariates into design matrix. Hypergeometric tests were performed to evaluate the concordance between each pair of gene lists [76]. GSEAs were performed by using GO and KEGG datasets. GO enrichments from UniProt [77] were checked using *clusterProfiler* package [78], *enrichGO* function. KEGG pathway enrichments were performed using *fgsea* package [79]. 

### 4.3. Co-Expression Network Analysis

Co-expression networks are suited well to reveal genes with similar regulation patterns across modules, which are highly connected gene sets, and ideal for making biological interpretations. Here, co-expression networks were built using combined AD and control data normalised for batch effects and based on Spearman correlations between genes (Dar S1). The genes involved in the top 1% of correlations by correlation coefficient were accepted for downstream analysis. Spearman correlation coefficients were converted to adjacency matrices and used to build undirected networks using the *igraph* package [80]. 

Using the random walk algorithm, densely connected subgraphs, henceforth called modules, were determined. Modules with a node size ≥ 20 and transitivity (clustering coefficient) > 0.5 were chosen for downstream analysis. Modules complying with these criteria from each network were matched to modules from other tissues and tested for significant overlaps by hypergeometric tests, which we used to indicate consistency between them and the presence of key genes within them. Then, differentially expressed gene sets were matched to these overlapping modules to understand whether co-expressed genes in these modules follow differential expression patterns, which was also tested by hypergeometric test. Next, we inspected the overlap of modules overlapping to gene set to see the coherence between tissues. We merged modules detected from each tissue. We narrowed genes of interest to hub genes (top 10% in terms of degree) assuming the higher probability of them being key genes or associated with them. GSEA was performed on them to obtain enriched biological annotations. Then, we looked at consistently shared hub genes among modules and searched for enriched metabolic pathways from HMR3 database. 

We consulted the Interactome Atlas from the Human Reference Protein Interactome Mapping Project [81] to identify PPI partners of the key genes from co-expression analysis and their family members. For this purpose, we downloaded HuRI, HI-union, Test space screens-19 and Lit-BM tables, which have in total 96,370 interactions between 20,209 genes. We converted ENSEMBL IDs to gene symbols in reference to GENCODE v27. It must be noted that interactomes from these tables only show binary protein interactions, being blind to additional interaction partners and indirect associations in protein complexes. Even more, database is estimated to cover only a low proportion of human binary PPIs, hypothetically millions, and cellular function of them are mostly unknown; therefore, we evaluated PPIs as mostly complementary and informative rather than explanatory for results. Genes of interest were investigated in HPA to find out their normal protein and gene expression levels in brain tissues compared to other organs and their biological annotations.

### 4.4. Genome-Scale Metabolic Modelling and Reporter Metabolite Analysis 

For each of DLPFC, TCX, and CBE datasets, we calculated AD and control consensus TPMs for each gene by taking arithmetic means across samples, without filtering out lowly expressed genes. Context-specific GEMs were constructed for each diagnosis group and for each brain region by overlaying group consensus TPMs onto HMR3 reference human model (8160 reactions and 3819 genes) and providing a list of 57 metabolic tasks that are compulsory for cellular growth. Modelling was performed on MATLAB v2020b (MathWorks Inc, USA), using tINIT algorithm in RAVEN 2.3.1 [82] toolbox for model construction and GUROBI (Gurobi Inc, TX) and MOSEK (Mosek ApS, Denmark) as the optimization solvers. 

Constructed metabolic models were compared based on three aspects. First, the general reaction content similarities were compared, namely using Hamming distance. This shows the similarities of diagnosis groups on metabolite level. Second, the differences in subsystem (i.e., metabolic pathway) coverage were shown for subsystems with at least 10% difference in one or more GEMs to show pathways significantly changed between conditions or tissues. Third, the functionality of GEMs was compared to perceive the differences in their metabolic activity.

RMA shows the metabolites around which transcriptional changes occur significantly between different conditions by the use of DEA results (Datasets S6–S8). The method is sensitive to small perturbations and easily reveals patterns in metabolic network [83]. We applied the RAVEN implementation of RMA on DESeq2 results for each brain region, i.e., log2 fold changes in AD group compared to control group and *p*-values for these changes. We focused on significant metabolites with *p*-value ≤ 0.05 and evaluated metabolites reported from down-regulated and up-regulated genes separately. 

## Figures and Tables

**Table 1:** Hypergeometric test results on the intersection of DEGs of datasets found by DESeq2.

VS	*p*-Val (Hyper)	Overlap	Region_1 DEG Size	Region_2 DEG Size	Both CodingGene Size
RO-MT	0.00054	128	2885	477	14,001
MC-RO	0.00516	347	2885	1515	14,186
MT-MC	9.20599 × 10^−11^	98	477	1515	14,186

RO: ROSMAP DLPFC, MT: MayoRNAseq TCX, MC: MayoRNAseq CBE. *p*-val is the *p*-value of hypergeometric test.

**Table 2:** Gene symbols of all shared DEGs, synthesised proteins and the most associated KEGG pathways for them. (**¥**) up-regulated in DLPFC and TCX, down-regulated in CBE, (#) up-regulated in all, (*) down-regulated in all.

Gene Symbol	Associated Protein Name (UNIPROT)	Most Associated KEGG Pathways
**ADAMTS2 ^¥^**	A disintegrin and metalloproteinase with thrombospondin motifs 2	-
**ATP1B3 ^#^**	Sodium/potassium-transporting ATPase subunit beta-3	Secretion (insulin, salivary, bile, gastric acid, pancreatic, aldosterone) has04961_Endocrine_and_other_factor-regulated_calcium_reabsorption has04973_Carbohydrate_digestion_and_absorption has04260_Cardiac_muscle_contraction has04978_Mineral_absorption
**BTG ^#^**	B-cell translocation gene 1 protein	has03018_RNA_degradation
**DAXX ^#^**	Death domain-associated protein 6	has04010_MAPK_signaling_pathway, has04210_Apoptosis, has05012_Parkinson_disease, has05014_Amyotrophic_lateral_sclerosis, has05022_Pathways_of_neurodegeneration, has05168_Herpes_simplex_virus_1_infection
DYNC2LI1 *	Cytoplasmic dynein 2 light intermediate chain 1	has04962_Vasopressin-regulated_water_reabsorption, has05132_Salmonella_infection
**FAM129B ^#^**	Protein Niban 2	-
**FAM167B ^#^**	Protein FAM167B	-
FAM90A1 *	Protein FAM90A1	-
**FBXO2 ^#^**	F-box only protein 2	has04068_FoxO_signaling_pathway, has04120_Ubiquitin_mediated_proteolysis, has04141_Protein_processing_in_endoplasmic_reticulum, has05132_Salmonella_infection
**GARNL3 ^#^**	GTPase-activating Rap/Ran-GAP domain-like protein 3	-
**GIT1 ^#^**	ARF GTPase-activating protein GIT1	has04144_Endocytosis, has04810_Regulation_of_actin_cytoskeleton, has05120_Epithelial_cell_signaling_in_Helicobacter_pylori_infection
**H2AFV ^#^**	Histone H2A.V	-
**HEBP2 ^#^**	Heme-binding protein 2	-
**ID3 ^#^**	DNA-binding protein inhibitor ID-3	has04350_TGF-beta_signaling_pathway
**NHEJ1 ^#^**	Non-homologous end-joining factor 1	has03450_Non-homologous_end-joining
**NT5DC2 ^#^**	5’-nucleotidase domain-containing protein 2	-
**PAFAH1B3 ^#^**	Platelet-activating factor acetylhydrolase IB subunit alpha1	has00565_Ether_lipid_metabolism
PLAGL1 *	Zinc finger protein PLAGL1	-
**RAF1 ^#^**	RAF proto-oncogene serine/threonine protein kinase	CENTRAL Signaling (JAK/STAT, TNF, VEGF, Insulin, Apelin, cAMP, mTOR etc.) Cancer (colorectal, pancreatic, breast, glioma, melanoma etc.) Infection (Hepatitis, Influenza, Tuberculosis, Salmonella etc.) has04210_Apoptosis, has04218_Cellular_senescence, has04510_Focal_adhesion, has04540_Gap_junction, has04935_Growth_hormone_synthesis, secretion_and_action has04726_Serotonergic_synapse, has04510_Focal_adhesion
**RALBP1 ^#^**	RalBP1-associated Eps domain-containing protein 2	has04014_Ras_signaling_pathway, has05212_Pancreatic_cancer
**S100A4 ^#^**	Calvasculin/Metastasin	-
**S100A6 ^#^**	Calcyclin/Growth factor-inducible protein 2A9	-
**SEPT9 ^#^**	Septin-9	-
**SMAD4 ^#^**	Mothers against decapentaplegic homolog 4	Signalling (Fox0, Wnt, apelin etc.), Cancer (colorectal etc.) has04110_Cell_cycle, has04520_Adherens_junction
**STARD10 ^#^**	START domain-containing protein 10	-
**STAT5B ^#^**	Signal transducer and activator of transcription 5B	Signalling (AGE-RAGE, JAK/STAT etc.), Myeloid leukemia has04217_Necroptosis, has04659_Th17_cell_differentiation
TRIM45 ^*^	Tripartite motif-containing protein 45	-
TRIM66 ^*^	Tripartite motif-containing protein 66	-
**TRIP10 ^#^**	Cdc42-interacting protein 4	has04910_Insulin_signaling_pathway
TTC14 ^*^	Tetratricopeptide repeat protein 14	-
**UBAP1 ^#^**	Ubiquitin-associated protein 1	-
UBXN8 ^*^	UBX domain-containing protein 8	has04141_Protein_processing_in_endoplasmic_reticulum
ZNF334 ^*^	Zinc finger protein 334	has05168_Herpes_simplex_virus_1_infection
**ZNF639 ^#^**	Zinc finger protein 636	-

**Table 3:** All shared DEGs and associated pathways *.

Pathway	Shared DEGs in the Pathway			
**hsa03450_Non-homologous_end-joining**	NHEJ1			
hsa04010_MAPK_signaling_pathway	DAXX	RAF1		
**hsa04012_ErbB_signaling_pathway**	RAF1	STAT5B		
hsa04014_Ras_signaling_pathway	RAF1	RALBP1		
hsa04015_Rap1_signaling_pathway	RAF1			
hsa04022_cGMP-PKG_signaling_pathway	ATP1B3	RAF1		
hsa04024_cAMP_signaling_pathway	ATP1B3	RAF1		
hsa04062_Chemokine_signaling_pathway	RAF1	STAT5B		
**hsa04068_FoxO_signaling_pathway**	RAF1	SMAD4		
hsa04071_Sphingolipid_signaling_pathway	RAF1			
hsa04072_Phospholipase_D_signaling_pathway	RAF1			
hsa04150_mTOR_signaling_pathway	RAF1			
hsa04151_PI3K-Akt_signaling_pathway	RAF1			
hsa04261_Adrenergic_signaling_in_cardiomyocytes	ATP1B3			
**hsa04210_Apoptosis**	DAXX	RAF1		
hsa04310_Wnt_signaling_pathway	SMAD4			
**hsa04350_TGF-beta_signaling_pathway**	ID3	SMAD4		
hsa04370_VEGF_signaling_pathway	RAF1			
**hsa04371_Apelin_signaling_pathway**	RAF1	SMAD4		
hsa04390_Hippo_signaling_pathway	SMAD4			
**hsa04550_Signaling_pathways_regulating_pluripotency_of_stem_cells**	RAF1	ID3	SMAD4	
hsa04625_C-type_lectin_receptor_signaling_pathway	RAF1			
hsa04630_JAK-STAT_signaling_pathway	RAF1	STAT5B		
**hsa04659_Th17_cell_differentiation**	SMAD4	STAT5B		
hsa04660_T_cell_receptor_signaling_pathway	RAF1			
hsa04662_B_cell_receptor_signaling_pathway	RAF1			
hsa04664_Fc_epsilon_RI_signaling_pathway	RAF1			
hsa04722_Neurotrophin_signaling_pathway	RAF1			
**hsa04910_Insulin_signaling_pathway**	RAF1	TRIP10		
hsa04912_GnRH_signaling_pathway	RAF1			
hsa04915_Estrogen_signaling_pathway	RAF1			
**hsa04917_Prolactin_signaling_pathway**	RAF1	STAT5B		
**hsa04919_Thyroid_hormone_signaling_pathway**	RAF1	ATP1B3		
hsa04921_Oxytocin_signaling_pathway	RAF1			
hsa04926_Relaxin_signaling_pathway	RAF1			
**hsa04933_AGE-RAGE_signaling_pathway_in_diabetic_complications**	SMAD4	STAT5B		
**hsa04935_Growth_hormone_synthesis,_secretion_and_action**	RAF1	STAT5B		
hsa05120_Epithelial_cell_signaling_in_Helicobacter_pylori_infection	GIT1			
**hsa05161_Hepatitis_B**	RAF1	SMAD4	STAT5B	
**hsa05200_Pathways_in_cancer**	RAF1	RALBP1	SMAD4	STAT5B
**hsa05210_Colorectal_cancer**	RAF1	SMAD4		
**hsa05212_Pancreatic_cancer**	RAF1	RALBP1	SMAD4	
hsa05213_Endometrial_cancer	RAF1			
hsa05215_Prostate_cancer	RAF1			
hsa05219_Bladder_cancer	RAF1			
**hsa05220_Chronic_myeloid_leukemia**	RAF1	SMAD4	STAT5B	
**hsa05221_Acute_myeloid_leukemia**	RAF1	STAT5B		
**hsa05223_Non-small_cell_lung_cancer**	RAF1	STAT5B		
hsa05224_Breast_cancer	RAF1			
**hsa05226_Gastric_cancer**	RAF1	SMAD4		

* Pathways in which shared DEGs are present significantly based on hypergeometric test. **bold:** signalling pathways and cancer-associated pathways.

**Table 4:** Hub genes shared by modules from different tissues and most associated ontologies.

Tissues	Hub Genes (Top 10%)
**DLPFC, TCX, CBE**	AMIGO1, GPRASP2
**DLPFC, TCX**	Cytoskeleton and its organisation ACTR3B, GABARAPL1, MARK1, NDEL1 Synaptic activity/plasticity AP2M1, ARHGEF9, CALM3, DLG3, GABRB3, L1CAM, NPTN Intra Golgi and retrograde Golgi-to-ER traffic AP3M2, ARF3, CFAP36, KIFAP3, KLC1, NAPB, NSF, RAB6B Ubiquitin/Proteasome System DNAJC5, HSPA12A Glucose Metabolism/Oxidative phosphorylation ATP6V1A, ATP6V1B2, HK1, SEH1L, SLC25A14, SLC25A4, SLC9A6 Other ATL1, B4GAT1, BTRC, C1orf216, CDK14, CDK5R1CHN1, CISD1, CLSTN3, CNTNAP1, EID2, FAM234B, FAM49A, GLS, GOT1, GPI, GUCY1B3, INPP4A, JAZF1, MAGEE1, MAPK9, MLTT11, MOAP1, MYCBP2, NDFIBP1, NDRG3, NELL2, NMNAT2, OPCML, PCMT1, PFN2, PHACTR1, PNMA2, PNMAL1, PPPIR7, PPP3CB, PPP3R1, PREP, PREPL, PRKCE, RBFOX2, REEP1, REEP5, RTN3, SEPT6, SMAP2, SNAP91, SV2B, SYT13, TMEM246, TSPYL1, UBE2O, UBFD1, VDAC1, VDAC3, WDR7, YWHAG, YWHAZ
**TCX, CBE**	Cytoskeleton and its organisation PAK1
**DLPFC, CBE**	Glucose Metabolism/Oxidative phosphorylation ATP6V1E1 Cytoskeleton and its organisation CDC42, DCTN2 Other ERLEC1, MPP1

**Table 5:** Descriptive statistics of GEMs.

	DLPFC-AD	DLPFC-Control	TCX-AD	TCX-Control	CBE-AD	CBE-Control
**Number of Reactions**	5727	5773	5895	5845	5898	5826
**Number of Metabolites**	4529	4593	4588	4541	4603	4542
**Number of Genes**	2494	2516	2632	2615	2585	2592

## Data Availability

Transcriptome data and relevant metadata are available in the AMP-AD Synapse platform. RNAseq harmonisation study is accessible by syn9702085. For ROSMAP study, refer to syn10498765, syn21323366 and syn3191090, syn3191087. For the MayoRNASeq study, refer to syn3817650, syn10498758 and syn5223705. The list of 57 metabolic tasks compulsory for cellular growth is available at https://github.com/sysmedicine/phd2020/tree/master/GEM/data as common_tasks_growth_RPMI1640 (Accessed 3 June 2021). The list of 256 metabolic tasks for humans is available at https://github.com/SysBioChalmers/Human-GEM/tree/master/data/metabolicTasks/ as metabolicTasks_Full (Accessed 3 June 2021).

## Supplementary Materials

The following are available online at https://www.mdpi.com/article/10.3390/ijms222111556/s1.
