# Supplementary material for: Revealing the Molecular Mechanisms of Alzheimer’s Disease Based on Network Analysis
Source: Int J Mol Sci. 2021 Oct 26;22(21):11556. doi: 10.3390/ijms222111556 (PMC8584243; doi:10.3390/ijms222111556)
Supplement: Supplementary file 1 [file ijms-22-11556-s001.zip › Bayraktar_SuppFigures.pdf]

Figure S1 Visualization of sample distributions from DLPFC based on highly variant genes and using UMAP to determine batch covariates

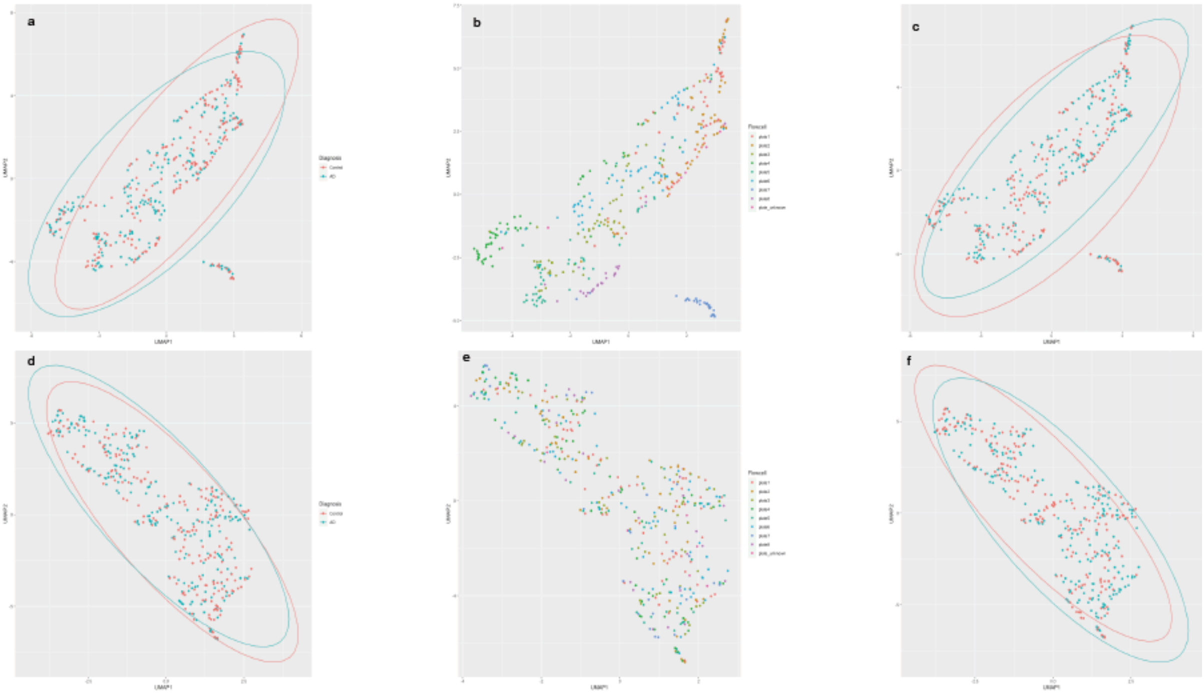

a-f: Visualization of sample distributions from DLPFC based on highly variant genes and using UMAP to determine batch covariates. Top row plot represents sample distributions before batch removal, and bottom row plots are for after removing batch covariate (flow cell). Samples in each plot were highlighted according to their diagnostic status, flow cell or source, respectively from left to right.

Figure S2 Visualization of sample distributions from TCX based on highly variant genes and using UMAP to determine batch covariates

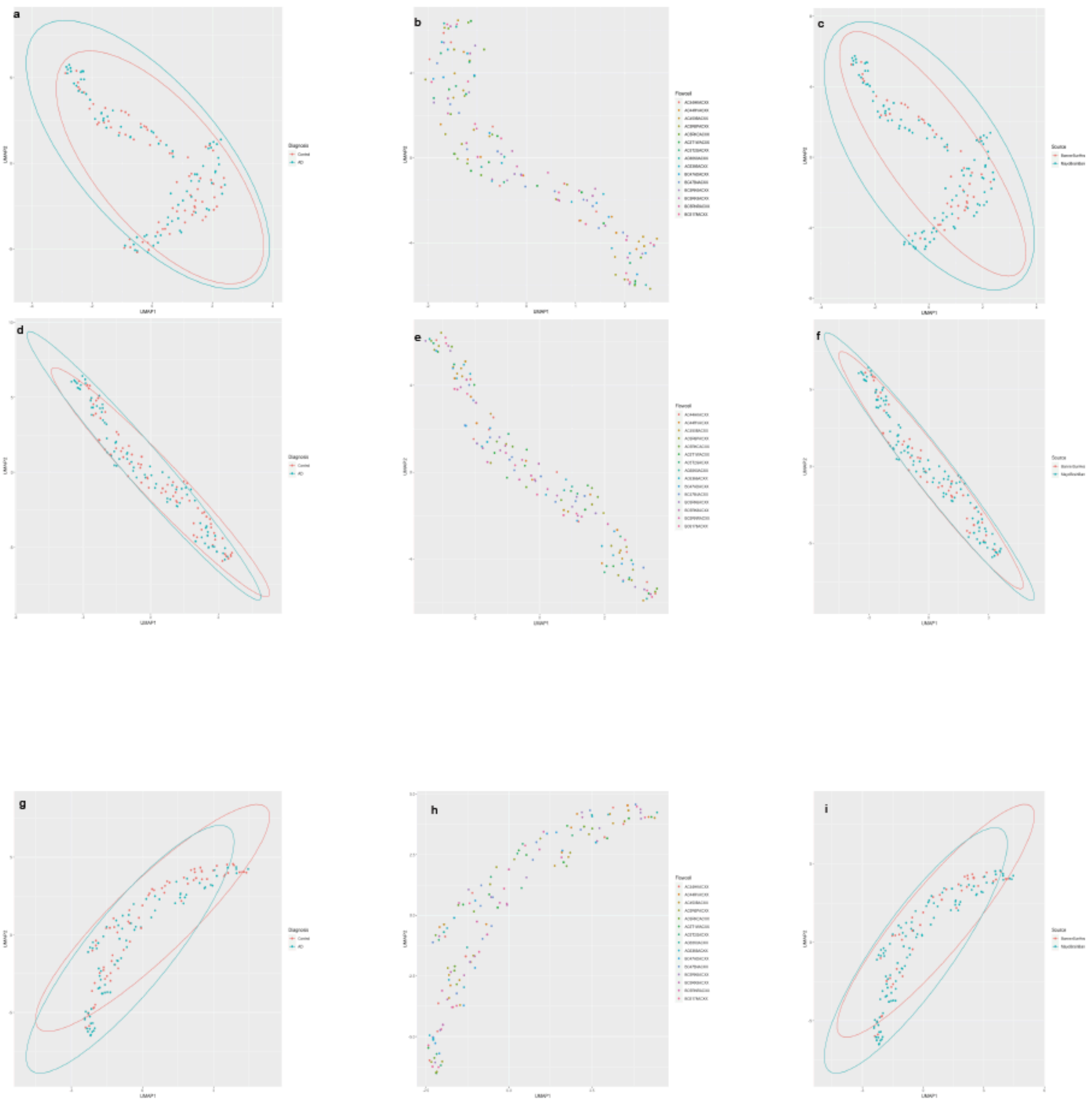

a-f: Visualization of sample distributions from TCX based on highly variant genes and using UMAP to determine batch covariates. Top row plots represent sample distributions before batch removal, and bottom row plots are for after removing batch covariate (flow cell). Samples in each plot were highlighted according to their diagnostic status, flow cell or source, respectively from left to right.
g-i: Visualization of sample distributions from TCX based on highly variant genes and using UMAP to determine batch covariates. Plots represent sample distributions after removing batch covariate (source). Samples in each plot were highlighted according to their diagnostic status, flow cell or source, respectively from left to right.

Figure S3 Visualization of sample distributions from CBE based on highly variant genes and using UMAP to determine batch covariates

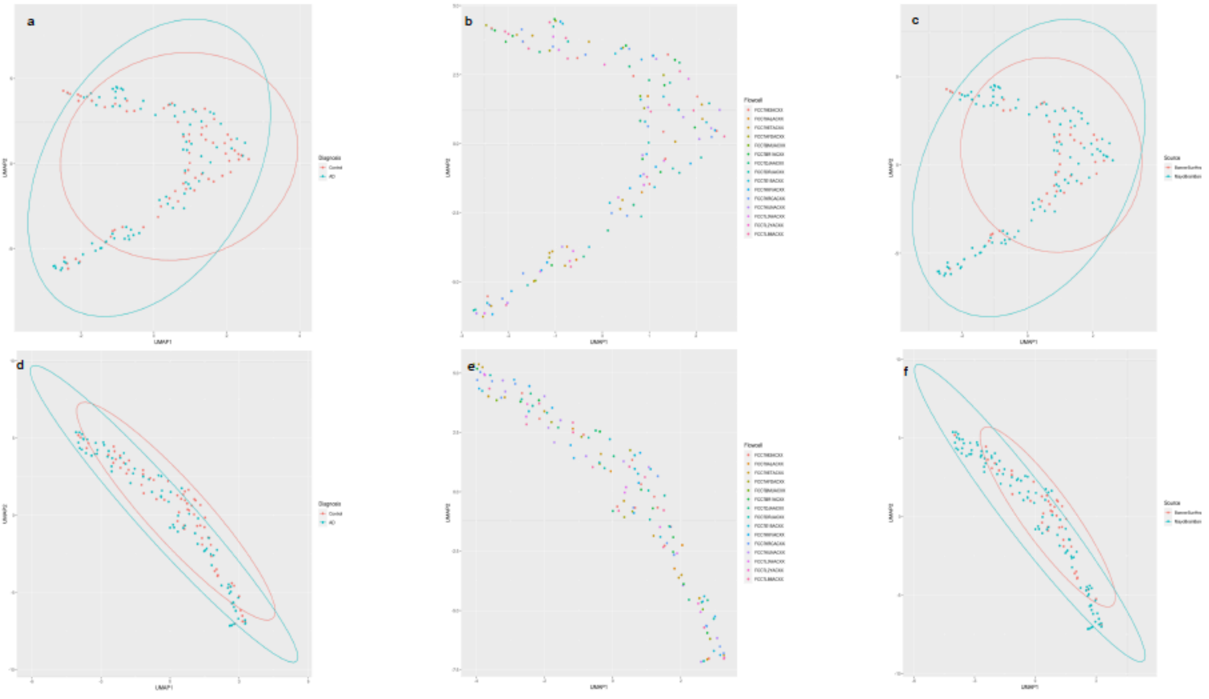

a-f: Visualization of sample distributions from CBE based on highly variant genes and using UMAP to determine batch covariates. Top row plots represent sample distributions before batch removal, and bottom row plots are for after removing batch covariate (source). Samples in each plot were highlighted according to their diagnostic status, flow cell or source, respectively from left to right.

Figure S4 Dispersion plots for ROSMAP DLPFC, MayoRNAseq TCX and CBE data sets

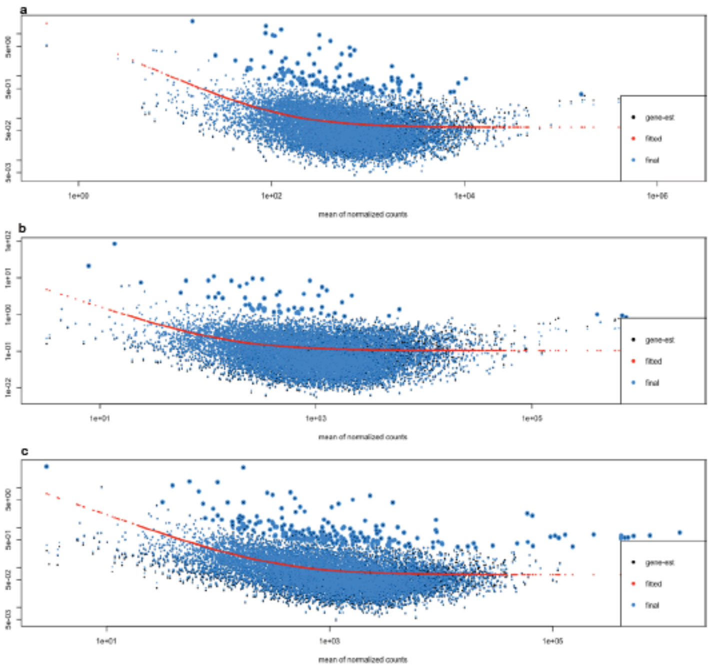

Figure S5 Significantly enriched or reduced KEGG pathways for differentially expressed genes in DLPFC and CBE, which were differentially expressed in other tissues

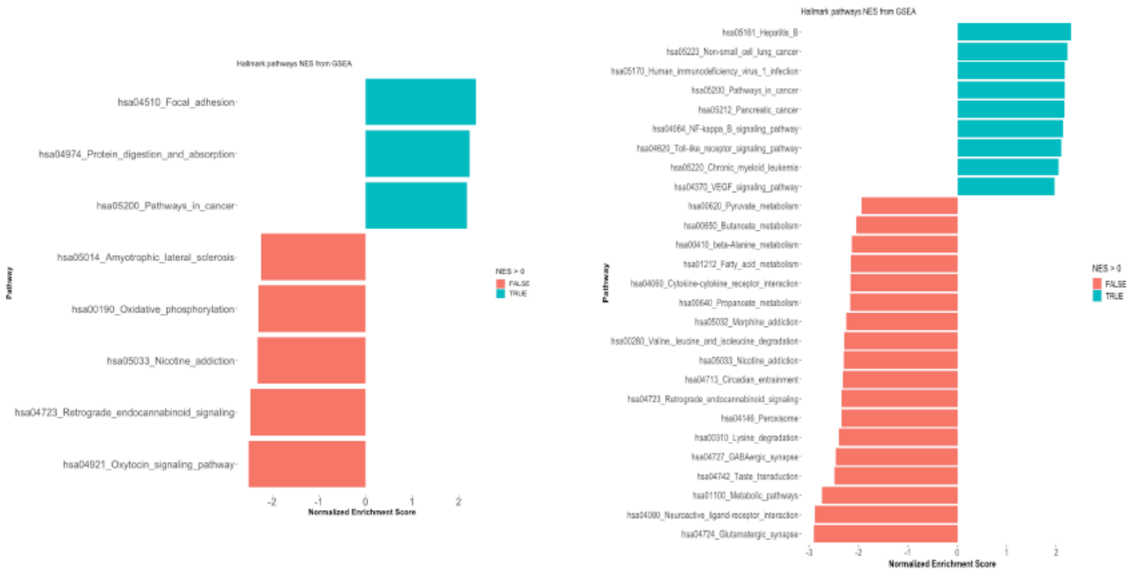

Significantly enriched or reduced KEGG pathways for differentially expressed genes in (left) DLPFC and (right) CBE, which were differentially expressed in other tissues (left: DLPFC-TCX, DLPFC-CBE, DLPFC-TCX-CBE; right: CBE-TCX, CBE-DLPFC, DLPFC-TCX-CBE)

Figure S6 Significant over-representations of Gene Ontology terms for DEGs from brain regions

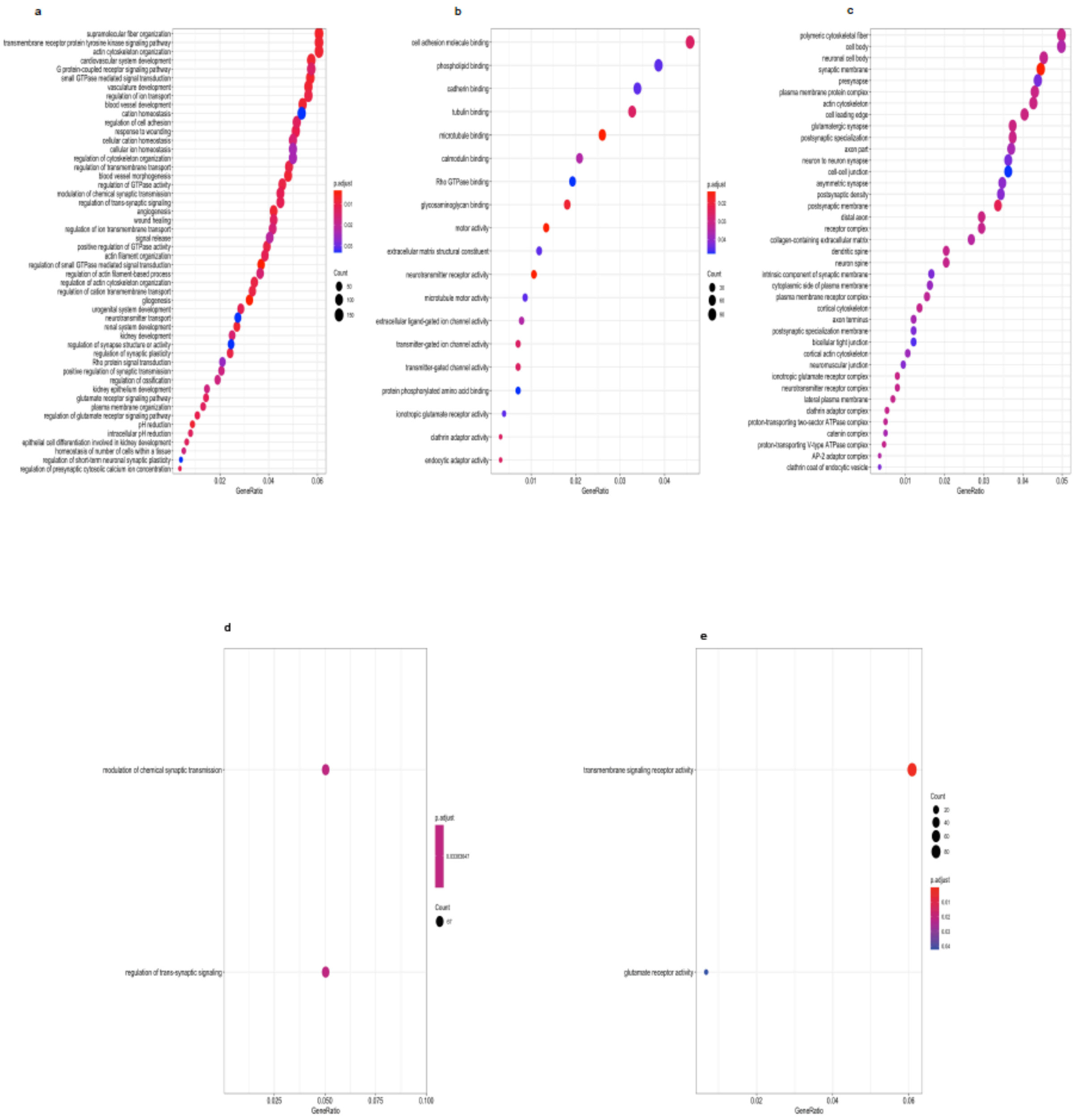

a-c: Significant over-representations of Gene Ontology terms for DEGs from DLPFC. Left to right: biological processes, molecular functions, cellular components.

d,e: Significant over-representations of Gene Ontology terms for DEGs from CBE. Left to right: biological processes, molecular functions.

Figure S7 Significant over-representations of Gene Ontology terms for hub genes from brain regions

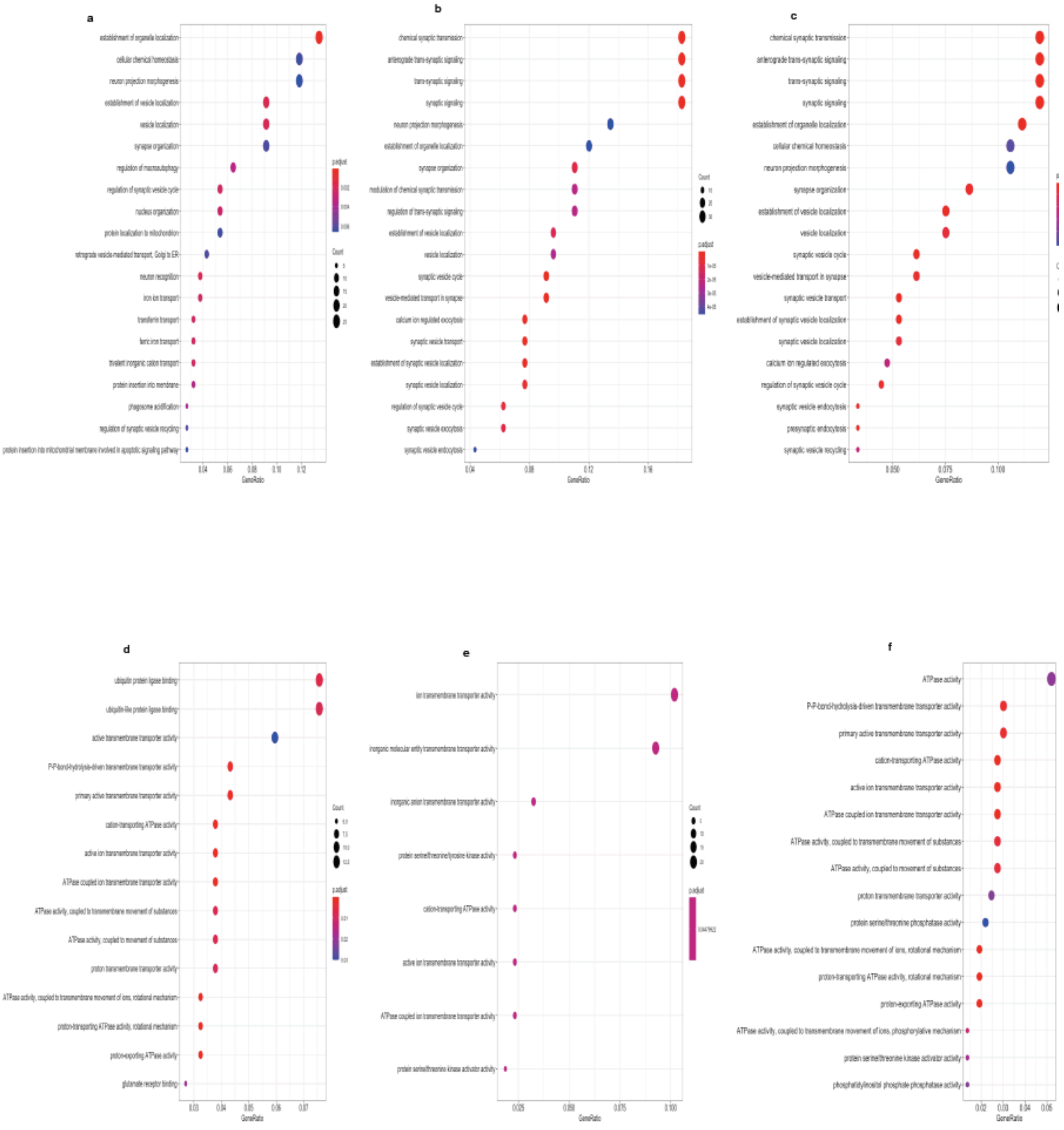

a-c: Significant over-representations of Gene Ontology biological processes for (left) hub genes from DLPFC, (middle) for hub genes from TCX and (right) for union of hub genes
d-f: Significant over-representations of Gene Ontology molecular functions for (left) hub genes from DLPFC, (middle) for hub genes from TCX and (right) for union of hub genes
g-i: Significant over-representations of Gene Ontology cellular components for (left) hub genes from DLPFC, (middle) for hub genes from TCX and (right) for union of hub genes

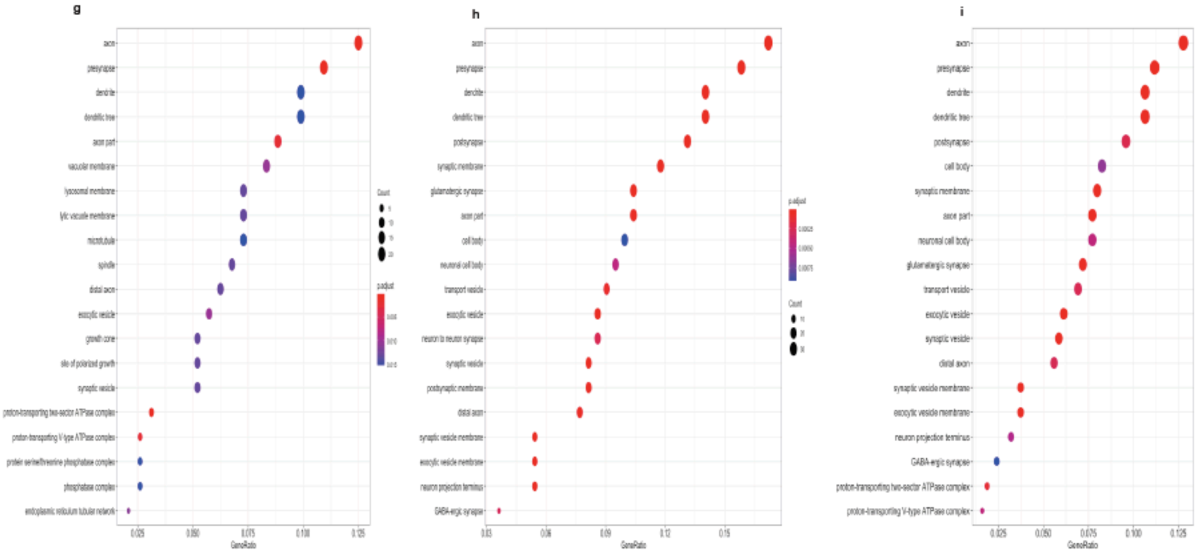
g
axon
presynapse
dendrite
dendritic tree
axon part
vacuolar membrane
lysosomal membrane
lytic vacuole membrane
microtubule
spindle
distal axon
exocytic vesicle
growth cone
site of polarized growth
synaptic vesicle
proton-transporting two-sector ATPase complex
proton-transporting V-type ATPase complex
protein serine/threonine phosphatase complex
phosphatase complex
endoplasmic reticulum tubular network
0.025
0.050
0.075
0.100
0.125
GeneRatio
Count
p.adjust
h
axon
presynapse
dendrite
dendritic tree
postsynapse
synaptic membrane
glutamatergic synapse
axon part
cell body
neuronal cell body
transport vesicle
exocytic vesicle
neuron to neuron synapse
synaptic vesicle
postsynaptic membrane
distal axon
synaptic vesicle membrane
exocytic vesicle membrane
neuron projection terminus
GABA-ergic synapse
0.03
0.06
0.09
0.12
0.15
GeneRatio
p.adjust
Count
i
axon
presynapse
dendrite
dendritic tree
postsynapse
cell body
synaptic membrane
axon part
neuronal cell body
glutamatergic synapse
transport vesicle
exocytic vesicle
synaptic vesicle
distal axon
synaptic vesicle membrane
exocytic vesicle membrane
neuron projection terminus
GABA-ergic synapse
proton-transporting two-sector ATPase complex
proton-transporting V-type ATPase complex
0.025
0.050
0.075
0.100
0.125
GeneRatio
